# Cellulose-Based Nanofibril Composite Materials as a New Approach to Fight Bacterial Infections

**DOI:** 10.3389/fbioe.2021.732461

**Published:** 2021-11-11

**Authors:** Somaye Rashki, Neda Shakour, Zahra Yousefi, Marzieh Rezaei, Mina Homayoonfal, Ehsan Khabazian, Fatemeh Atyabi, Fatemeh Aslanbeigi, Rouzita Safaei Lapavandani, Samaneh Mazaheri, Michael R Hamblin, Hamed Mirzaei

**Affiliations:** ^1^ Department of Microbiology and Immunology, Faculty of Medicine, Kashan University of Medical Sciences, Kashan, Iran; ^2^ Department of Medicinal Chemistry, School of Pharmacy, Mashhad University of Medical Sciences, Mashhad, Iran; ^3^ Student Research Committee, Faculty of Medicine, Mashhad University of Medical Sciences, Mashhad, Iran; ^4^ School of Allied Medical Sciences, Shahroud University of Medical Sciences, Shahroud, Iran; ^5^ Department of Medical Biotechnology and Nanotechnology, Faculty of Medicine, Mashhad University of Medical Sciences, Mashhad, Iran; ^6^ Research Center for Biochemistry and Nutrition in Metabolic Diseases, Institute for Basic Sciences, Kashan University of Medical Sciences, Kashan, Iran; ^7^ Department of Pharmaceutics, Faculty of Pharmacy, Tehran University of Medical Sciences, Tehran, Iran; ^8^ Nanotechnology Research Centre, Faculty of Pharmacy, Tehran University of Medical Sciences, Tehran, Iran; ^9^ School of Medicine, Kashan University of Medical Sciences, Kashan, Iran; ^10^ Student Research Committee, Kashan University of Medical Sciences, Kashan, Iran; ^11^ Department of Gynecology and Obsteterics, Shaheed Beheshti University of Medical Sciences, Tehran, Iran; ^12^ Department of Analytical Chemistry, Faculty of Chemistry, University of Kashan, Kashan, Iran; ^13^ Laser Research Centre, Faculty of Health Science, University of Johannesburg, Doornfontein, South Africa

**Keywords:** cellulose, nanofibrils, antimicrobial activity, bacterial infections, nanotechnology

## Abstract

Antibiotic resistant microorganisms have become an enormous global challenge, and are predicted to cause hundreds of millions of deaths. Therefore, the search for novel/alternative antimicrobial agents is a grand global challenge. Cellulose is an abundant biopolymer with the advantages of low cost, biodegradability, and biocompatibility. With the recent growth of nanotechnology and nanomedicine, numerous researchers have investigated nanofibril cellulose to try to develop an anti-bacterial biomaterial. However, nanofibril cellulose has no inherent antibacterial activity, and therefore cannot be used on its own. To empower cellulose with anti-bacterial properties, new efficient nanomaterials have been designed based on cellulose-based nanofibrils as potential wound dressings, food packaging, and for other antibacterial applications. In this review we summarize reports concerning the therapeutic potential of cellulose-based nanofibrils against various bacterial infections

## Introduction

Infections caused by antibiotic-resistant bacteria are now a serious worldwide challenge to public health, causing unacceptable morbidity, mortality, and expense to society. For several decades, the number of infections associated with antibiotic resistant bacteria has steadily increased (Wang, Hu et al., 2017). Consequently, novel methods must be devised to destroy microbes in a more environmentally friendly manner. Towards this goal, nanomaterials have been widely explored to generate new antimicrobial agents (Martins, Freire et al., 2012). Nanoscale materials have shown great promise for reducing the growth of antibiotic resistance in general (Zille, Almeida et al., 2014). However, the development of antimicrobial materials is faced with multiple problems. The development of suitably biodegradable and biocompatible surfaces with prolonged anti-bacterial activity, at a comparatively low cost has not yet been achieved (Council 2006; Tavakolian, Jafari et al., 2020). Cellulose is an abundant biopolymer widely used as a reinforcing component in fiber-based thermo-plastic composite materials. Cellulose is regarded as an almost unlimited natural resource, which could help to meet the increasing demand for more biocompatible and biodegradable products. Cellulose is a natural biopolymer, that can take the form of cellulose nanofibers (CNF) or fibrils varying from 50 to 60 nm in diameter. These can be used to generate nanostructured paper sheets, thin films, multifunctional nanocomposites, or translucent films (Kalia, Dufresne et al., 2011; Moohan, Stewart et al., 2020; Zeng, Zeng et al., 2021). These structures show low gas-barrier properties, along with other advantages (Abe, Iwamoto et al., 2007). The use of naturally derived nano-biopolymers can reduce their possible toxicity and widen their applicability. However, it has been found that cellulose in its natural state does not possess any intrinsic antibacterial activity. Nevertheless, it does possess an abundance of functional groups that may be chemically modified for a variety of applications (Tavakolian, Jafari et al., 2020). These materials can have powerful and long-lasting antimicrobial activity, which is resistant to leaching within the biological environment. In fact, their unique advantage over other antibacterial materials results from the non-leaching action, where the bacteria are required to be in direct contact with the surface (Hassan, Forsman et al., 2020). Nevertheless, such materials have been considered to be useful alternatives in situations where this direct contact occurs automatically, such as water purification, anti-bacterial filters, food packaging, wound dressings, and anti-bacterial coatings used in health care environments to avoid cross-infection between patients and hospital spread. This review discusses the therapeutic potential of cellulose-based nanofibrils against various bacterial species and infections. Also, in this review, we summarize the properties and anti-bacterial activity of cellulose, and the preparation of cellulose-based nanofibrils.

## Cellulose Properties and Anti-Bacterial Activity

**FIGURE 1:**
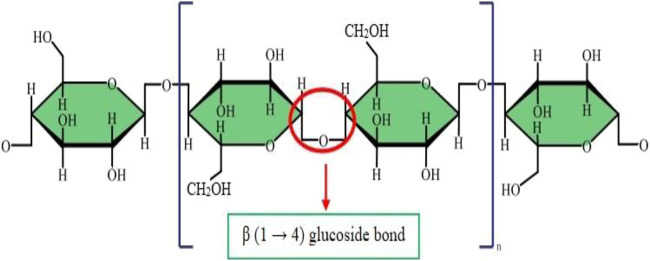
Molecular structure of cellulose.

**FIGURE 2:**
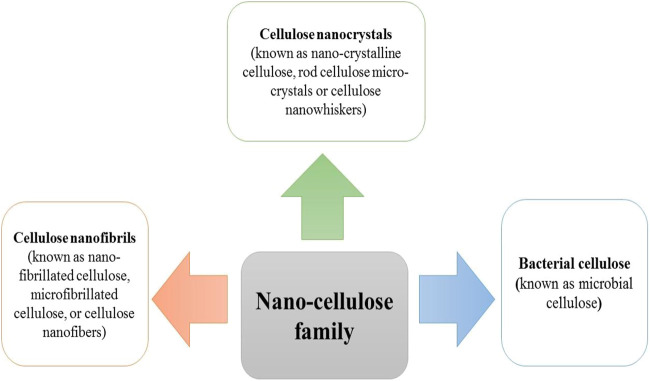
The family of nano-cellulose structures.

As mentioned earlier, cellulose is the most plentiful organic material on the planet earth. Nearly a billion tons of cellulose is produced from different plants annually (Kafy, Kim et al., 2017). Cellulose is composed of a linear polymer chain made up of glucose monomers with the formula (C_6_H_10_O_5_)_n_ (Figure 1) and called poly-β-1,4-linked anhydro-D-glucose. Moreover, the linear cellulose chains can form supramolecular assemblies by formation of hydrogen bonds between neighboring hydroxyl groups of adjacent cellulose chains. Therefore, cellulose can produce a regular networked macrostructure. The growth of nanotechnology has led to the investigation of nanocellulose as a new versatile biomaterial (Lin and Dufresne 2014). Nanocellulose is produced from natural renewable sources, such as plants (wood and grasses) as well as bacterial cellulose. Nanocellulose contains repeating β-1,4 linked d-glucose units, each with three active hydroxyl groups at the C2, C3, and C6 positions of the glucopyranose ring (Li, Cha et al., 2018). The nano-cellulose family may be divided into three broad groupings of materials (Ullah, Manan et al., 2021) (Figure 2).

**FIGURE 3:**
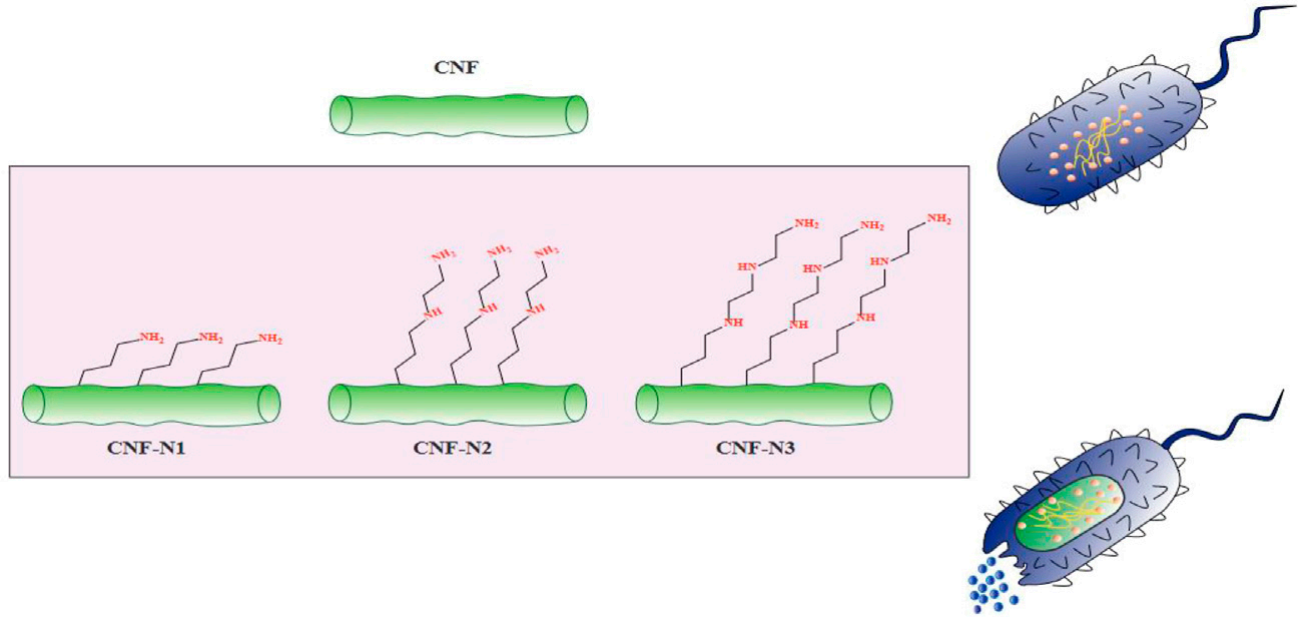
The antibacterial activity of aminoalkyl grafted CNF. This figure adapted from Saini et al. (Saini, Belgacem et al., 2017).

One growing application of CNFs is in the development of novel antibacterial materials based on cellulose. In contrast to other natural bio-polymers such as chitosan (CH), that exhibit intrinsic antimicrobial activity, cellulose is not considered to be intrinsically antimicrobial (Tavakolian, Okshevsky et al., 2018). Hence, cellulose requires to be chemically modified to become an effective antimicrobial agent. Because there are multiple reactive hydroxyl groups on the surface of cellulose fibrils, they can react with carboxylic acids, amines, or aldehydes, to change the properties of the polymer (Tavakolian, Jafari et al., 2020). There are four main methods for the preparation of antimicrobial materials based on nanocellulose, including modifying the surface, adding antibiotics, combining with other nanomaterials, or combining with other antibacterial agents (Saini, Belgacem et al., 2015; Anirudhan and Deepa 2017; Singla, Soni et al., 2017). For instance, chemical attachment of aminoalkyl groups to the cellulose surface promoted the lethal activity of cellulose against *S. aureus* (Saini, Belgacem et al., 2017)*.* Moreover, the antibacterial activity of CNF was increased by increasing the alkyl chain length of the aminoalkyl groups (Figure 3). Besides the alkyl chain length, increasing the number of amino groups can also improve the antibacterial activity (Kim, Choi et al., 1997). In one study, Jiang et al., functionalized cellulose by the introduction of N-halamines (chloro- or bromoamines) to the CNC backbone via reaction with the hydroxyl groups (Jiang, Qiao et al., 2017). The mechanism of action of N-halamines is the direct reaction of halogen atoms such as Br− or Cl− with the amino or thiol groups of the proteins present on the bacterial cells, leading to inactivation or growth inhibition (Kocer, Akdag et al., 2008). In addition, aldehyde-modified nanofibrillated cellulose showed antibacterial activity against MRSA (methicillin-resistant *S. aureus*). The action mechanism for aldehyde-modified nanofibrillated cellulose was to increase the acidic environment for the bacteria due to the presence of dialdehyde groups (Tavakolian, Jafari et al., 2020). Antimicrobial materials based on nanocellulose can also be employed in other applications, such drug carriers, packaging materials, and wound dressings (Li, Cha et al., 2018).

Recently, polymers extracted from plants, such as pectin and cellulose, have attracted much attention for the synthesis and modification of metal oxide nanoparticles (MONPs) and metal nanoparticles (MNPs). The antibacterial and anti-infective properties of nanoparticles (NPs) can be enhanced using the antimicrobial properties of primary and secondary metabolites obtained from these herbal sources. Among the MONPs/MNPs investigated, Ag, CuO, ZnO, and Cu NPs have exhibited more potent antimicrobial properties due to their ability to produce reactive oxygen species (ROS) (Alavi and Kennedy 2021). The encapsulation and loading of AgNPs by cellulose nanostructures can decrease the side effects of argyrosis or argyria, sometimes observed in patients. In one comparison study, 10 wt% and 5 wt% of AgNP/TiO_2_ NPs were loaded onto cellulose acetate (CA) nanofibers (CNFs) to produce CA/TiO_2_/AgNP-2 and CA/TiO_2_/AgNP-1, respectively. The order of antibacterial activity and growth inhibition after 24 h incubation for both *E. coli* and *S. aureus* were as follows: CA/TiO_2_/AgNP-2 ˃ CA/TiO_2_/AgNP-1 ˃ CA/TiO_2_ ˃ CA (Kim et al., 2011; Jatoi et al., 2019b). The precise bacterial strain and the physicochemical properties of the MNP/MONP may influence the antibacterial activity. CuNPs and AgNPs were prepared respectively by chemical reduction and ultraviolet radiation methods, followed by immobilization onto CNFs. Potent bactericidal activity against *B. subtilis* (Gram-positive bacteria) and *E. coli* (Gram-negative bacteria) was reported for Cu/CNFs and Ag/CNFs, respectively (Phan, Dorjjugder et al., 2019). Chemical (CD) and mechanical (MD) techniques were used to deposit AgNPs onto ZnONPs to form nanocomposites (NCs). The process was followed by capping the NCs with carboxymethyl cellulose (CMC), to produce CMC-Ag-ZnO NCs. More potent antibacterial properties were reported for Ag-ZnO NCs (CD) compared to AgZnO NCs (MD). For example, MRSA showed 16 and 18 mm zones of inhibition for Ag-ZnO NCs (MD) and Ag-ZnO NCs (CD), respectively (Lungu, Vasile et al., 2016).

## Preparation of Cellulose-Based Nanofibrils

Currently, nanofibrillated cellulose can be produced from several different cellulosic resources. Most studies have used wood as the most important industrial natural resource for production of cellulosic fibers (Missoum, Belgacem et al., 2013). It is possible to employ different techniques for extracting the cellulose nanofibers from wood and other bulk cellulose sources.

### Biological Methods

Biological techniques can be used to disrupt the crystalline structure of the cellulose nanofibrils, for example enzyme-assisted hydrolysis (Hayashi and Kondo 2015), as well as mild mechanical methods (Iwamoto, Kai et al., 2010), or a combination of these two treatments (Hubbe, Rojas et al., 2008). Enzymatic hydrolysis is an effective technique that uses a purified endoglucanase to promote defibrillation. It results in the specific hydrolysis of the glucosidic bonds along the cellulose chain, mainly in less crystalline domains (Moon, Martini et al., 2011). Lignin, cross-linked polysaccharide networks, and glycosylated proteins are all components of plant cell walls. Lignin can reinforce hemicellulose and cellulose microfilms, just like steel rods firmly implanted in concrete. Pretreatment can reduce this reinforcement, thus biomaterials, chemicals or enzymes can be more easily in contact with each other and therefore able to generate more effective products. The pretreatment process can improve the directly accessible surface area (ASA), thereby increasing the cellulose availability. Different sub-steps are involved with different pretreatments, which can improve the overall digestion. However, the higher accessibility of the cellulose can change the physical structure and chemical composition. This preparation of nanocrystals from bacterial cellulose does not involve dangerous chemical reagents, so it is a green production method (Zhang, Barhoum et al., 2019).

Microbial sources of CNC, including fungi, bacteria, and other microorganisms, provide fibers with an individual diameter of 2 nm, which form a nanofiber bunch or bundle with a diameter of approximately 100 nm. These fibers have useful inherent properties with a crystallinity percentage around 84–89% (Thomas, Paul et al., 2011).

One of the main challenges in the formulation of nanocellulose structures is the need to combine hydrophilic nanocellulose with hydrophobic polymers, leading to problems with mixing of the components and aggregation. Therefore, there will be a possibility of forming a non-dispersed matrix structure, with the loss of the reinforcing nanoscale properties. The hydrophobic nature of the nanocellulose composite may be circumvented in the presence of polar organic solvents like N-methyl pyrrolidone, dimethyl sulfoxide, or N,N-dimethyl formamide (Dufresne 2013). Low yields of products with a wide range of sizes have been reported for existing techniques. Hence, there is a need to improve on these methods for large scale industrial purposes or commercialization. Pre-treatment techniques have often been suggested as the best strategies for greater efficiency. The production of nanocellulose products using enzymes is often carried out in industrial processes. The production of nanocellulose using enzymatic treatments is so important that it has motivated many researchers to study this approach. The production of nanocellulose using enzymatic hydrolysis is a multi-step process. The first step involves the pretreatment of the lignocellulosic biomass. The second and most important step involves controlled enzymatic hydrolysis of the fibers. The third step consists of homogenization of the enzyme-treated materials by washing, suspension, or mechanical homogenization. Fabricated nanocellulose products can be prepared with a uniform consistency using these optimized methods (Ribeiro, Pohlmann et al., 2019). The drawbacks of the enzymatic hydrolysis method are the longer time and expensive enzymes that are needed (Ribeiro, Pohlmann et al., 2019). Accordingly, research laboratories and industrial companies have focused in recent years on reducing these costs by improving the saccharification efficiency, increasing the permeability of the bulk material, and reducing environmental pollution.

### Mechanical Methods

**FIGURE 4:**
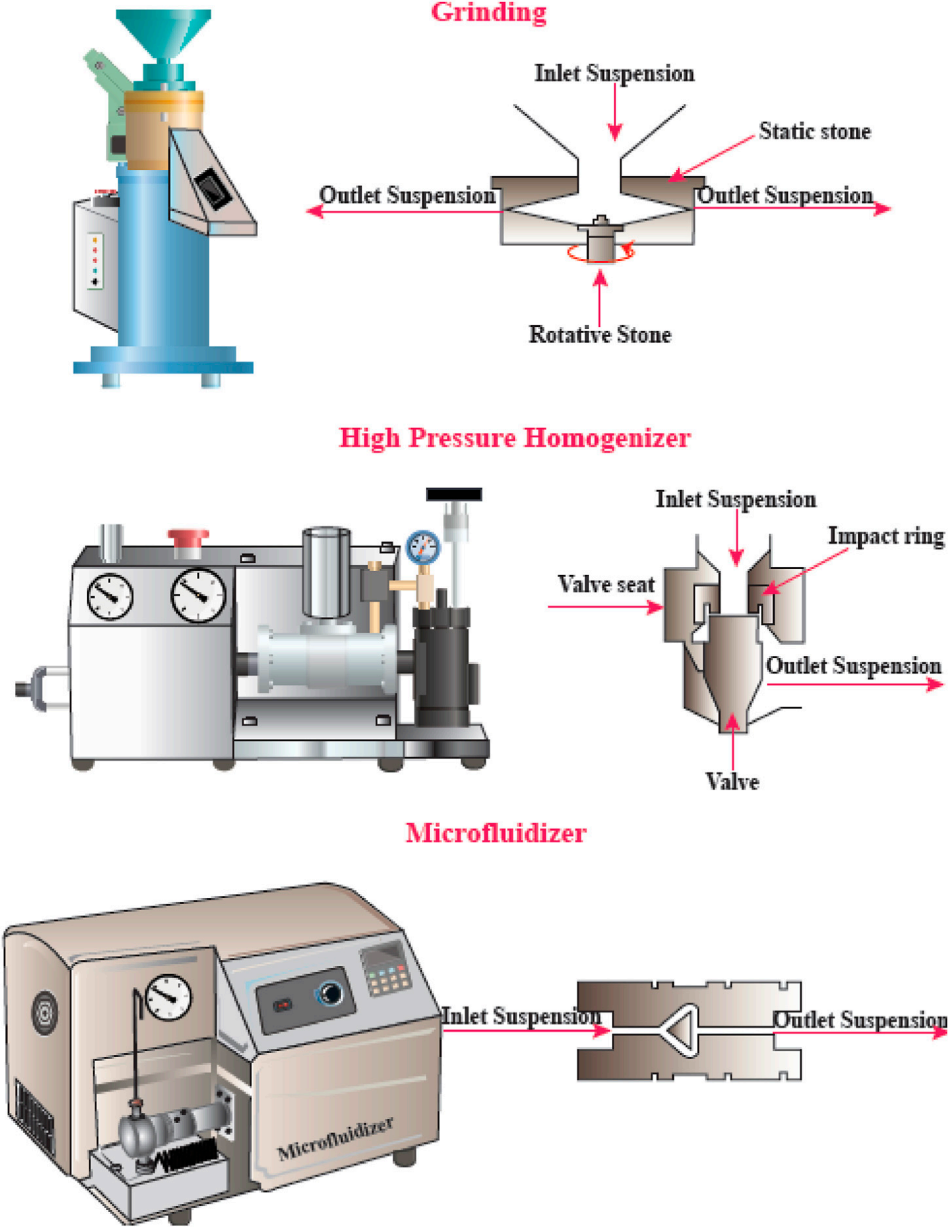
Mechanical methods for CNF production. This figure adapted from www.niro-soavi.com, www.microfluidicscorp.com and www.masuko.com.

Physical or mechanical extraction procedures include high-pressure homogenization, cryocrushing, microfluidization, and grinding (Figure 4). The fibers produced by these procedures have low stability and a variable ratio of length to diameter due to the application of higher fiber tension (Lavoine, Desloges et al., 2012). The other drawback of the procedure has been proposed to be its relatively unpredictable effects. Depending on the mechanical treatment and the level of mechanical force, the breaking of hydrogen bonds between the adjacent fibrils can occur. Mechanical methods can be costly (materials and instrumentation), have lower effectiveness, and consume more energy than chemical methods. A chemical pretreatment can decrease the consumption of energy and can also increase the surface hydrophobicity. Furthermore, mechanical treatments can decrease the DP (degree of polymerization) and polymer length from 1,200–1,400 to between 850 and 500 glucose monomers. Moreover, the DP can affect the nanofiber tensile resistance that may be nearly as high 2 Gpa (Rojas, Bedoya et al., 2015).

#### High-Pressure Homogenization (HPH)

The HPH procedure involves passing a cellulose suspension through a narrow nozzle at high pressure into a container. The high pressure and velocity and the impact produce shear forces within the fluid, causing shear rates to increase and reducing the size of the fibers to the nanoscale. The advantages of HPH are its simplicity, high efficiency, and no need for organic solvents. The first application of the HPH process to generate NFC from wood pulp was reported in 1983 (Abdul Khalil et al., 2014).

#### Microfluidizer

**FIGURE 5:**
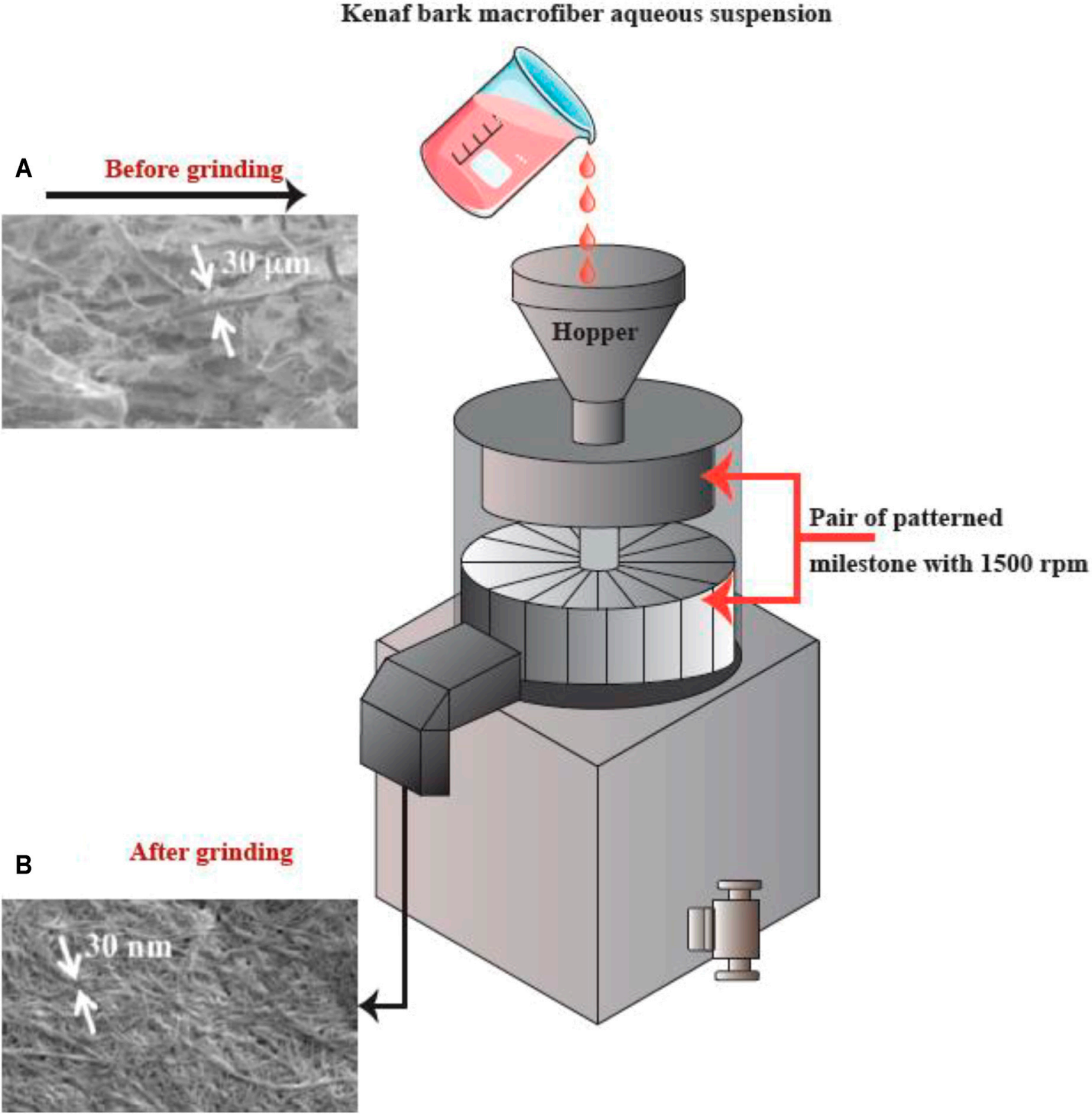
The apparatus for the microfluidizer method, and SEM images of the fibers after treatment. This figure adapted from Nishino et al. (Nishino 2017).

A progressively higher velocity can be obtained when the cellulose suspension is passed at high pressure through geometrically designed microchannels (Figure 5). Therefore, researchers employed a narrow Z-shaped enclosure with decreasing dimensions of 400–200–100 µm for fibrillation production. The lower the dimensions of the enclosure, the greater the degree of fibrillation. At the end of this process, a heat exchanger is employed to cool the product stream to the environmental temperature (Missoum, Belgacem et al., 2013).

#### Grinding

**FIGURE 6:**
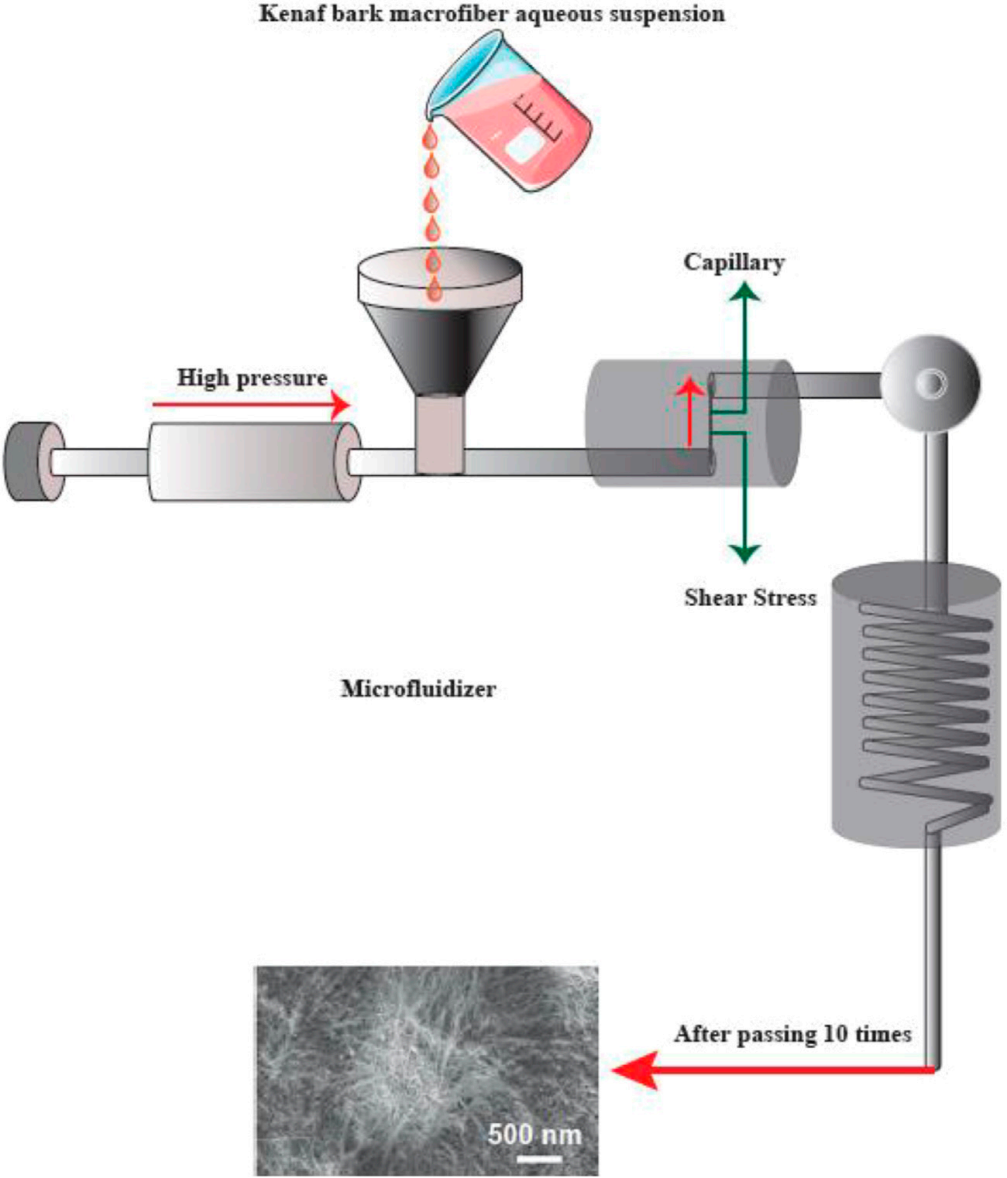
The apparatus for grinding, and SEM images **(A)** prior to and **(B)** following the grinding into refined macro-fibers (scale bar: A. 1,000 μm and B. 1 μm). This figure adapted from Nishino et al. (Nishino 2017).

Grinding is another method that can be used for breaking up cellulose into nanofibers (Figure 6). The grinding equipment consists of a rotating and a static grindstone, where a slurry of wood pulp is pumped between both the grindstones. Therefore, the mechanism of fibrillation by grinding, is the break-down of the structure of the cell walls and the interchain hydrogen bonds through shear force which converts the pulp to nanofibers (Abdul Khalil et al., 2014).

#### Cryocrushing

In this procedure, the cellulosic fibers are swollen in water, and then submerged in liquid nitrogen, followed and afterward are shattered by pestle and mortar. The application of force on the frozen cellulosic fibers caused the rupture of the cell walls, because of the extra pressure exerted by the ice crystals thus producing the nanofibers. In this regard, Wang and Sain generated nanofibers from soybean waste products by a high pressure defibrillation process followed by cryocrushing (Abdul Khalil et al., 2014; Rojas, Bedoya et al., 2015).

### Chemical and Mechanical Methods

Chemical-mechanical methods are the commonest procedures employed for manufacturing nanofibers, with a higher ratio of length to diameter, and smaller diameters compared to those from purely mechanical processes. In this approach, the non-cellulosic content is mostly eliminated by alkali treatment or acid hydrolysis. Comparing the disadvantages and advantages of the nanofiber extraction procedures shows that chemomechanical techniques have been the most successful for nanofiber production and purification (Zhang, Elder et al., 2007).

## Cellulose-Based Nanofibrils Against Bacterial Infections

Carboxymethyl cellulose (CMC) has become a widely used carbohydrate biopolymer in food packaging and biomedical applications, because of its good film-forming ability, wide availability, non-toxicity, and renewability. CMC films provide a suitable barrier against the environment, with good mechanical properties and transparency. Nevertheless, researchers have investigated many approaches to further improve the mechanical and barrier features, such as blending with other bio-degradable lipids or polymers, and introducing plasticizers, fillers, and cross-linking agents. The use of nanofillers can improve the physical characteristics of the biopolymers, and can also introduce antimicrobial and antioxidant properties to the packaging films (Ahmadi, Ghanbarzadeh et al., 2019). Some of the nanofillers that have been incorporated into biopolymer matrixes are; titanium dioxide (TiO_2_) (Mehrabani et al., 2018a; Karimian et al., 2020), zinc oxide (ZnO) (Oun and Rhim 2017), silicon dioxide (SiO_2_) (Song, Luo et al., 2013), silver (Mehrabani et al., 2018b; Karimian et al., 2020), and copper oxide (Ashjari, Dorraji et al., 2018). Nanoparticles (NPs) composed of chitin have been added to cellulose nanofibers (CNF) and cellulose nanocrystals (CNC) (Lizundia, Fortunati et al., 2016; Tang, Zhang et al., 2018). In recent times bioactive natural compounds have been preferred over synthetic anti-microbial agents or other functional materials to avoid health problems and environmental hazards (Jung and Zhao 2016). For example, *Salvadora persica* L (Meswak) is a small shrub from the Salvadoraceae family, which is indigenous to the Middle East is found in the south of Iran, Saudi Arabia, Africa, and India (Halawany 2012). Traditionally, Miswak root has been employed as a toothbrush, and was recommended as a “chewing stick” for oral hygiene by the FAO/WHO in 1989. The biological activity of *S. persica* stick extract (SPE) includes antioxidant activity (Mariod, Matthäus et al., 2009), and SPE contains numerous bioactive inorganic and organic compounds, like salvadourea, saponin, salvadorine, vitamin C, amides, cyanates, tannins and alkaloids. Moreover, SPE contains glycosides (salvadoraside and salvadoside), terpenoids, resins, sterols, flavonoids, terpenes, and fatty acids (stearic, oleic, and linoleic acids). Inorganic components include sodium chloride, potassium chloride, calcium, manganese, phosphorus, silica, fluoride, sulfur and magnesium (Al-Sohaibani and Murugan 2012; Farag, Fahmy et al., 2017; Noumi, Snoussi et al., 2017). Researchers have investigated the antimicrobial effects of SPE against Gram-negative and Gram-positive bacteria, including *Staphylococcus*, *Streptococcus*, *Mycobacteria*, *Escherichia coli*, as well as *Candida ssp*. a well-known oral yeast (Al-Bayati and Sulaiman 2008; Fallah, Fallah et al., 2015; Al-Ayed, Asaad et al., 2016).

Ahmadi et al. prepared an antimicrobial nanocomposite based on CNF, SPE, and CMC (Ahmadi, Ghanbarzadeh et al., 2019). SEM (scanning electron microscopy) was used to visualize the CNF distribution within the CMC matrix, while EDX (energy dispersive X-ray) spectroscopy showed the presence of minerals and sulfur-containing compounds in the nanocomposite. XRD (X-ray diffraction) and FTIR (Fourier transform infrared) spectroscopy confirmed the presence of SPE and CNF in the nanocomposite structure. Incorporation of CNF increased the UTS (ultimate tensile strength) while it reduced EB (elongation at breakpoint). However, the addition of 200 or 400 mg/mL resulted in an EB increase without any decrease in the UTS. TGA (thermal gravimetric analysis) showed that SPE promoted the thermal stability of the nanocomposite. The pure SPE nanocomposites showed good antibacterial activity against both *Staphylococcus aureus* (Gram-positive) and *Escherichia coli* (Gram-negative) bacteria. The CMC/CNF-5%SPE nanocomposite showed improved thermophysical properties and a better UV-barrier function. The encouraging antimicrobial activity against both Gram-positive and Gram-negative bacteria, suggests that adding an extract of *S. persica* to CNF composite films could produce a biologically active food packaging.

Skin wounds are often infection-prone and difficult to treat, and improved wound dressings are urgently required (Balakrishnan, Mohanty et al., 2005). Conventional dressings can be synthetic or natural bandages, cotton wool, or gauze. Saline and gauze help the wound healing in the initial stages due to absorption of exudates and blood, and allow debridement and cleansing. However, these dry dressings do not provide sufficient moisture in the wound environment, and, they tend to become sticky as fluid production decreases. This means they are difficult to remove, resulting in patient discomfort. Due to the lack of occlusion allowing moisture evaporation, gauze dressings also result in a dehydrated wound bed. Therefore, new dressings have been developed to improve upon traditional wound healing treatments. The essential characteristic of modern dressings is the ability to retain and provide enough moisture in the wound environment to facilitate the healing process. Modern dressings are principally categorized based on their materials, including alginates, hydrogels, and hydrocolloids, which can be formed into gels, foam sheets, or thin films (Radhakumary, Antonty et al., 2011). Natural polymers such as glycolipids, polysaccharides, proteoglycans, peptides, and proteins have been widely applied in regenerative medicine. Due to their biodegradability, biocompatibility, and similarity to the extracellular matrix, natural polymers have been applied in wound and burn dressings. Natural polymers help the damaged tissue to repair itself, and result in improved skin regeneration via stimulating cellular healing processes (Mogoşanu and Grumezescu 2014).

Many researchers have attempted to prepare improved and innovative wound dressings. In fact, wound dressings based on hydrogels offer several benefits like a moist environment, a cooling sensation, and allow gaseous exchange, and absorption of the wound exudate (Cheng, Liu et al., 2017; Zhao, Wu et al., 2017). The major cause of wound infections is external contamination by bacterial pathogens, which can not only delay wound healing, but can also invade the host tissue leading to sepsis and possibly to death. Antibiotics and antiseptics are the most often used procedure for prevention of bacterial wound infections. To minimize the of use of broad spectrum antibiotics which can cause antibiotic resistance, researchers have used AgNPs (silver NPs) as local anti-bacterial agents with less potential to cause resistance and fewer adverse events (Dhand, Soumya et al., 2016). The action mechanism of AgNPs includes causing damage to the bacterial cell membranes, and formation of reactive oxygen species (ROS) (Marambio-Jones and Hoek 2010). However, the toxicity of AgNPs may limit their use, because the particles can penetrate into the tissue and release Ag + ions. The above problem can be solved by stabilizing the AgNPs by preparing composites with diverse materials (Loo, Fane et al., 2013). The surface of bacterial cellulose was embellished with self-assembled AgNPs to supply anti-bacterial activity (Wu, Zheng et al., 2014). The slower release of silver ions decreased the host cytotoxicity, and decreased bacterial growth over the long term. Bandages constructed from AgNP-decorated nanofibers showed a powerful antibacterial activity against *S. aureus*, and showed lower cytotoxicity against keratinocytes in an *ex vivo* skin model. Furthermore, antibacterial hydrogels have been prepared using acrylic acid polymers combined with cellulose for full-thickness wound healing. These hydrogels not only killed bacteria, but encouraged cell proliferation and migration (Loh, Mohamad et al., 2018).

Ji Un Shin et al. prepared an antibacterial hydrogel from AgNP-immobilized alginate combined with CNFs obtained from tobacco-based cellulose nanofibers (TCNF) (Shin, Gwon et al., 2018). The CNF surface hydroxyl groups were oxidized to carboxylate groups by reacting with TEMPO ((2, 2, 6,6-tetramethyl piperidine-1-yl) oxidanyl). This was then treated with silver nitrate solution for surface absorption of silver ions. Spectroscopic analysis, XRD, and electron microscopy showed the immobilization of higher quantities of the AgNPs on the TCNF surface compared to the native CNF surface. The AgNP immobilized TCNF was combined with an alginate gel, and the Ag ions were released gradually from the matrix over 7 days. The alginate gels loaded with AgNPs demonstrated comparable antibacterial activity to alginate gels loaded with silver-ions, but showed lower cytotoxicity towards mammalian cells. The anti-bacterial gels could be used on different skin surfaces and wounds to inhibit bacterial growth without harming epidermal cells.

Titanium dioxide (TiO_2_) is a widely studied semi-conducting material used in photonic, optical, energy, and catalysis applications. TiO_2_ is best known as a photocatalyst, and exists in three different phases; brookite, anatase, and rutile (Gupta, Singh et al., 2013). TiO_2_ has been shown to possess photoactivated bactericidal properties, especially under blue or UV light. Under ultraviolet irradiation, TiO_2_ generates many ROS including hydroxl radicals, superoxide, and hydrogen peroxide that disrupt the cell walls of bacteria and inhibit their respiratory system (Mukhopadhyay, Basak et al., 2010; Verdier, Coutand et al., 2014). Nevertheless, because of the fast rate of electron-hole recombination within TiO_2_, the ROS production is limited (Nino-Martinez, Martinez-Castanon et al., 2008). Furthermore, the immobilization of noble metals (like silver) on the TiO_2_ surface, slows the recombination of electrons and holes, and therefore increases ROS generation (Seery, George et al., 2007; Liga, Bryant et al., 2011). Antibacterial nanocomposites including TiO_2_ can prevent bacterial colonization by the release of antibacterial agents, and creation of ROS causing oxidative stress (Wang, Li et al., 2005). A nanocomposite based on cellulose acetate (CA) including TiO_2_ and AgNPs, showed bactericidal effects based on ROS production (hydrogen peroxide, hydroxyl radicals, singlet oxygen, and superoxide) (Jatoi, Jo et al., 2018).

Jatoi et al. carried out AgNP immobilization on TiO_2_ nanoparticles using an environmentally green procedure, and combined the TiO_2_/AgNPs into a matrix of CA nanofibers (Kim et al., 2011; Jatoi et al., 2019a). The TiO_2_/AgNP nanocomposite particles were prepared by coating TiO_2_ nanoparticles with polydopamine hydrochloride followed by a treatment in AgNO_3_ solution. Subsequently, the TiO_2_/AgNP nanocomposites were added into the CA solution and electrospun to fabricate CA/TiO_2_/AgNP composite nanofibers. These samples were characterized using FTIR, XRD, XPS, TEM, SEM, and EDX, and their antibacterial activity investigated. The SEM images showed the regular morphology of the nanofibers, and the antibacterial tests showed significant bacterial killing for 36 h and significant bacterial growth inhibition for 72 h.

One challenge faced by wound dressings, especially for acute wounds, is the control of bleeding and hemorrhage, that is frequently a cause of trauma death (Gaston, Fraser et al., 2018). Numerous clinical hemostatic formulations have been investigated based on chitosan, thrombin, fibrin, or collagen but they might be difficult to store and use outside a hospital setting. Gelatin is readily available by hydrolysis of collagen, is low cost, and stable in storage, with good hemostatic effects (Lan, Lu et al., 2015; Chen, Guo et al., 2016).

Liu et al. designed a nanocomposite hydrogel with a green color, by combining gelatin (G) with Ag-NH2 NPs (aminated silver NPs) and CNF (Liu, Dai et al., 2018). An IPN (interpenetrating polymeric network) was created by this multi-component interaction, taking the form of a dynamic ionic-bridged (non-covalent) cross-linked hydrogel, CNF/G/Ag. Moreover, a hydrogel dressing containing Ag-NH2 NPs (0.5 mg/mL) showed antibacterial activity, self-recovery ability, good mechanical properties, the ability to absorb fluid from the wound bed (2093.9 g/m2 daily), and effective hemostatic performance. More importantly, evaluation of wound healing *in vivo* and *in vitro* with CNF/G/Ag demonstrated good biocompatibility (∼100% viability of infected cells) and wound healing ability (83.3% survival and ∼90% healed after 14 days). This hydrogel dressing based on cellulose may be useful for skin wound dressings using green materials.

Hydrogen peroxide (H_2_O_2_) is a reactive oxygen species (ROS) formed by oxidoreductase enzymes (lactoperoxidase, glucose oxidase and myeloperoxidase), which is capable of eliminating bacteria by inhibiting protein production, disrupting cell membranes and metabolism, oxidation of thiol groups, and depletion of ROS scavengers. Moreover, H_2_O_2_ can play a role as a substrate for halide or pseudohalide oxidation for the formation of stronger antimicrobial agents (Thallinger et al., 2013). Recently attention has been drawn to laccases, a copper-containing oxidoreductase enzyme found in plants and fungi, due to having no need for cofactors and the broad substrate specificity (Strong and Claus 2011). Laccase enzymes can catalyze reactions with natural polyphenols resulting in the formation of antimicrobial agents, which can kill Gram-negative and Gram-positive bacteria (Janusz, Pawlik et al., 2020). Bacterial nanocellulose (BNC) can form a useful ultrafine porous lattice for the immobilization of laccase because of its unique properties, such as high capacity for water absorption, high purity, three-dimensional nanostructure, and mechanical strength. The three-dimensional porous structure of BNC enables good access of substrates to the active site for continuous operation, easy recovery, and good penetration (Sulaiman, Mokhtar et al., 2015). In a recent study by Sampaio et al., the physical immobilization of a commercial laccase preparation onto BNC was evaluated to assess the antibacterial activity of laccase for wound dressing applications (Sampaio, Padrão et al., 2016). They reported that laccase immobilized onto BNC and formulated as a wound dressing showed satisfactory cytotoxicity for biomedical purposes, with antimicrobial activity against Gram-negative (26%) and Gram-positive (92%) bacteria. Depending on the volume of discharge, the dressing should be renewed on average every 1–3 days, and that laccase could act as an effective, non-toxic, inexpensive and durable antimicrobial dressing, even its stability was limited (Sampaio, Padrão et al., 2016).

Naturally occurring polymers such as proteins or polysaccharides are ofen incorporated into antimicrobial products and dressings. Cationic biopolymers alone (e.g. chitosan, CS) or in combination with cellulose, can act as biocides (Kupnik, Primožič et al., 2020). CS is a natural antimicrobial agent derived from chitin by partial deacetylation, and has attracted much attention in commercial fields. When CS is loaded onto nanocellulose (NC), it could enhance the mechanical properties of the NC and also increase the antimicrobial activity of cellulose-based compounds (Boroumand, Badie et al., 2021). CS is a natural polysaccharide containing amino-groups with unique features, including intrinsic antibacterial activity, non-toxicity, biocompatibility, and biodegradability (Rashki, Asgarpour et al., 2020). Poonguzhali et al. employed crystalline nanocellulose (CNC) to prepare antimicrobial hybrid structures containing CS. They fabricated CS polyvinylpyrrolidone (PVP)-NC (CPN) bionanocomposites for wound dressing applications (Poonguzhali, Basha et al., 2017). The biocompatible and non-toxic PVP has many applications in wound dressings and also in targeted drug delivery. Polymeric PVP films are effective drug delivery systems, in particular via the buccal and sublingual routes (Poonguzhali, Basha et al., 2017). A modified disk diffusion test was employed to determine the antibacterial activity of CPN against Gram-negative and Gram-positive bacteria. The results showed that the zones of inhibition were larger for *P. aeruginosa* (Gram-negative) compared to *S. aureus* (Gram-positive). The reason for the better inhibition of Gram-negative bacteria was attributed to an interaction between the positive charge of CS and the more negative charge of Gram-negative bacterial cell membrane, resulting in leakage from the bacterial cells and impairment in the microbial metabolism. Moreover, the large surface area of the NC could help the CS to bind to the bacteria, and increase the antimicrobial activity (Kupnik, Primožič et al., 2020).

Chitin, a β-1,4-N-acetylglucosamine linear polysaccharide, is part of the cell wall of fungal species, and the exo-skeleton of crustaceans and insects (Szymańska-Chargot, Chylińska et al., 2019). Because of its excellent crystallinity and robust inter-molecular bonds, chitin is non-reactive, but has poor water solubility. To improve its solubility, it can undergo partial deacetylation to form chitosan (CH). CH dissolves in water at lower pH and has been used to from composites with a broad range of other polymers (Deng, Jung et al., 2017). The principal drawback of chitosan composites is their poor mechanical characteristics (HPS, Abdul Khalil et al., 2016). The positive charge of CH has led to its use as a natural anti-microbial agent against a variety of microorganisms, with applications in food quality and safety (Kim, Min et al., 2011). Due to its antibacterial activity, biocompatibility, non-toxicity, and biodegradability, CH has been investigated in the food industry as a thickener and stabilizer, in biomedical engineering, and even in agriculture as a fertilizer (de Alvarenga 2011). The molecular structure of CH affects its anti-microbial activity, including its degree of polymerization (chain length), and its % deacetylation. Other important parameters include, the tested microorganisms, the type of medium, the microbial cell age, the chemical composition of the media especially the pH (Kong, Chen et al., 2010).

Szymańska-Chargot et al. prepared films composed of CCNF (carrot cellulose nanofibrils) combined with CHIT (low-viscosity CH) by vacuum filtration (Szymańska-Chargot, Chylińska et al., 2019). The amount of CH in the films varied between 9 and 33% of dry weight. The morphology of the film surface was examined by SEM, and they evaluated the CH distribution within the CCNF matrix. The bonds between CCNF and CHIT were confirmed by FTIR and they found a physical interaction between CHIT and CCNF instead of hydrogen bonding. This result explained the wettability by water. The addition of chitosan to the nanocellulose matrix increased the contact angle of water, so the surface of the composite became more lipophilic. The binding between chitosan and nanocellulose provided an overall denser structure. Analysis of the thermal properties showed better stabilization at high temperatures, with deterioration only occurring at >300°C. Moreover, the addition of CHIT to the CCNF matrix reduced the Young’s modulus (reduced from 14.71 GPa for CCNF to 8.76 GPa for CCNF/CHIT5). The composite tensile strength (the highest force to a create a fracture) was reduced following the addition of chitosan (from 145.83 MPa for CCNF to 129.43 MPa for CCNF/CHIT5). The composite showed a good inhibitory effect versus *S. epidermidis* and *E. coli*. *Micrococcus luteus* was inhibited at higher concentrations of chitosan in the composites, but *Candida krusei* and filamentous fungi were not inhibited Table 1 lists some cellulose-based nanofibrils that could be used in composites against bacterial infections.

**TABLE 1:** Various cellulose-based nanofibrils used against bacterial infections.

Cellulose nanofibril composite	Form	Target microorganisms	Model (In vitro/*In vivo*/Human)	Mechanism of antibacterial activity	Finding/application	Ref
Cellulose nanofibrils/PVA/AgNPs	Film	*Bacillus subtilis*	*In vitro*	Ag + ions release and permeation of bacterial cell membranes	Addition of AgNPs into CNF/PVA produced a composite with anti-microbial activity	Limaye, Gupta et al. (2019)
		*Escherichia coli*		AgNPs incorporation into CNF/PVA matrix allowed bacterial growth inhibition		
Carboxymethyl cellulose/cellulose nanofibril/*Salvadora persica*. L extract (CMC/CNF-/SPE)	Film	*Staphylococcus aureus ATCC29213*	*In vitro*	Cell wall destruction	Utilized for antimicrobial biomaterials	Ahmadi, Ghanbarzadeh et al. (2019)
		*E. coli ATCC 25922*		Change the membrane permeability Interact with bacterial outer membrane	Active food packaging	
		—		Change the membrane potential and integrity	Protects products from UV	
		—		Change the bacterial water osmosis gradient	—	
AgNP@TCNF	Hydrogel	*E. coli*	*In vitro*	Ag + sustained release for a prolonged period	Wound dressing	Shin, Gwon et al. (2018)
				Medium from hydrogel incubation decreased bacterial growth with no cytotoxicity	Prolonged antibacterial activity	
Poly (εCaprolactone)/Cellulose Nanofiber/ZrO2	Film	*S. aureus*	*In-vitro*	Antifungal and anti-bacterial activity due to nZrO2 in the composite	Wound dressing	Karimian, Mehrabani et al. (2020)
		*E. coli*		Good antibacterial activity		
		*Candida albicans*		—		
Cellulose/TiO2/Ag nanofibers	—	*E. coli*	*In vitro*	—	Applicable in bone tissue regeneration	Ashraf, Sofi et al. (2020)
		*S. aureus*			Wound-healing applications	
Cellulose nanofibrils/TiO_2_/antibiotics	—	*S. aureus NBRC 100910*	*In vitro*	—	Potentially useful as antimicrobial patches	Galkina, Önneby et al. (2015)
Cellulose nanofibrils/TiO2/Phos		*E. coli NCTC9001*		—	Long-term release of antibiotics	
—		—		—	Prolonged action to prevent bacterial growth	
—					Application as disinfectants	
AgNP/cellulose nanofibers	—	*E. coli*	*In vitro*	AgNPs provide slow release of Ag + ions	Biocomposite for antibacterial applications	Jatoi et al. (2019a)
		*S. aureus*		Complete inhibition of bacterial growth		
		—		Ag + ions inhibit ATP production, DNA synthesis, and inhibit respiratory system, causing bacterial killing		
Cellulose acetate (CA)/TiO2/AgNP	—	*E. coli*	*In vitro*	Disruption of bacterial cell walls	Nano-composite for prolonged anti-bacterial applications	Jatoi et al. (2019b), Kim et al. (2011)
		*S. aureus*		Inhibition of DNA synthesis and respiratory systems		
		—		ROS (reactive oxygen species) generation		
CA/carbon nanotube (CNT)/AgNP nanofibers	—	*E. coli*	*In vitro*	ROS generation	Long term antibacterial applications	Jatoi, Ogasawara et al. (2020)
		*S. aureus*		Disruption of bacterial cell walls		
		—		Damage to DNA		
		—		Inhibition of bacterial growth		
Cellulose nanofibrils/G/Ag	Hydrogel	*S. aureus*	*In vitro*	—	Wound dressing	Liu, Dai et al. (2018)
		*Pseudomonas aeruginosa*	*In vivo*			
Cu@SiO2/bacterial cellulose nanofibers	Film	*S. aureus*	*In vitro*	Bacterial inactivation	Suitable for biomedical applications	Ma, Huang et al. (2016)
		*Escherichia coli*		Interaction with microbial DNA inhibits replication	Prolonged antimicrobial activity	
		—		Destruction of sulfhydryl groups in the metabolic enzymes in the electron transfer chain	—	
		—		Binds to bacterial cell walls	—	
		—		Enhance membrane permeability Denature protein structure	—	
		—		Change enzyme activity	—	
		—		Cell death	—	
AgNPs/cellulose nanofiber (AgNP/CNF)	Aerogel	*E. coli*	*In vitro*	Release of Ag ions	Increased antifungal and antibacterial activity	(Matsuyama, Morotomi et al. 2019)
		*Aspergillus niger*		Oxidation of the AgNPs surface in contact with media		
		—		Ag species bind to microbial membrane increasing permeability		
		—		Inhibit replication and respiration		
		—		High surface area of CNF aerogel		
		—		Acts as a sponge to enhance antifungal and antibacterial activity		
		—		CNFs act as a sponge-like support for AgNPs		
EMPO-oxidized cellulose nanofibers (TOCNF)/PLA/TiO2	Film	*Bacillus subtilis*	*In vitro*	Addition of TiO2 to nanocomposite films improved antibacterial activity against Gram-positive and Gram-negative bacteria	Use as a packaging material	El-Gendy, Abou-Zeid et al. (2017)
		*P. aeruginosa*			Increased antibacterial effects	
		*E. coli*			—	
		*S. aureus*			—	
Carrot cellulose nanofibrils (CCNF)/chitosan	Film	*S. epidermidis*	*In vitro*	—	CCNF composite plus chitosan showed antimicrobial activity	Szymańska-Chargot, Chylińska et al. (2019)
		*E. coli*			Bacteriostatic and fungistatic	
		*B. cereus*			—	
		*Micrococcus luteus*			—	
		*Candida krusei*			—	
Cu/cellulose nanofibrils	—	*E. coli*	*In vitro*	Antibacterial activity was proportional to the release of copper or silver ions	Composite metal/cellulose nanofibers showed high antibacterial activity	Phan, Dorjjugder et al. (2019)
Ag/cellulose nanofibrils		*B. subtilis*				
Cellulose nanofibers/polylactic acid/ethanolic extract of propolis (CNFs/PLA/EEP)	Film	*S. aureus*	*In vitro*	Propolis acts via inhibiting replication of DNA and cell division	Higher antibacterial efficacy against Gram-positive bacteria	Abdulkhani, Hosseinzadeh et al. (2017)
		*B. anthracis*				
		*C. albicans*				
		*B. cereus*				
		*S. enterica*				
		*E. coli*				

## Future Perspectives

One major challenge faced by drug delivery systems is the poor solubility of the drug in water. Thus, to overcome this problem, formulation scientists have been searching for new appropriate excipients. Cellulose nanofibers are such a new excipient, and have only recently been investigated in the pharmaceutical context. The principal characteristics of cellulose nanofibers include lack of toxicity, useful surface chemistry, unique colloidal properties, high surface-area-to-volume, biodegradability, excellent mechanical and rheological properties, and gas barrier properties in the dry state. Recent studies have suggested there are positive molecular interactions between poorly-soluble drugs and cellulose nanofibers. also It has also been shown that in some cases, the interactions between drug molecules and cellulose nanofibers improved the apparent solubility of those drugs. The appropriate cellulose nanofibers can be assembled into a variety of structures, such as capsules, particles, Pickering stabilized lipophilic droplets, films, wet-stable foams, aerogels, and solid materials containing closed cells. These structures can result in tailored sustained release kinetics, and rapid release of poorly soluble drugs (Löbmann and Svagan 2017; Posada, Velásquez-Cock et al., 2020; Pandey 2021). One recent study described cellular solid materials containing closed cells that could be self-assembled using cellulose nanofibers to encapsulate poorly-soluble drugs. Due to their buoyant properties, these foams exhibited prolonged drug release characteristics (hours or even days), and could probably be used as a drug delivery system that could be retained within the stomach for site-specific drug release. However, a rapid release system could also be obtained by formulating aerogels consisting of films or nanoparticles containing poorly-soluble drugs with the appropriate cellulose nanofibers as the matrix material. Moreover, prolonged drug release from films over periods of months could be achieved by choosing different cellulose nanofibers (Moohan, Stewart et al., 2020). Cellulose nanofibers have a unique molecular arrangement that enables the stabilization of oil-droplets in aqueous media (Pickering stabilization). For example, lipid-based droplets could be used for delivery of poorly soluble drugs. Cellulose nanofibers are truly versatile excipients with regard to the regulation of drug release kinetics (Löbmann and Svagan 2017). Nevertheless, more research is needed to fully understand their interactions and establish their role in the delivery of poorly-soluble drugs both *in vitro* and *in vivo* in disease models.

Cellulose nanofibrils are frequently used in the formulation of antimicrobial materials, such as antibacterial hydrogels, wrapping papers, and thin films. These products can be used as drug carriers, food packaging films, infected wound-healing dressings, and multifunctional antibacterial films. Nevertheless, since the cellulose nanofibrils have little intrinsic antimicrobial activity, their application has been limited in biomedical applications. Hence, different technologies are used to improve the antibacterial properties of cellulose nanofibril-based materials, including antibiotic addition, surface modification, combining with nanomaterials, or antibacterial polymers. Various modifications have led to improvements in the physicochemical features of materials, particularly at the nanoscale (Li, Cha et al., 2018; Bagde and Nadanathangam 2019; Han, Wang et al., 2019; Wang, Yin et al., 2020). However, how the incorporation of exogenous molecules affects the biocompatibility and/or cytotoxicity of the cellulose nanofibrils should be addressed in future studies. Despite some remarkable advances having been made in biomedical cellulose nanofibrils, this field is still in its infancy.

## Conclusion

Since nanocellulose does not have any antimicrobial activity on its own, its use as antimicrobial wound dressings is limited. Therefore, anti-microbial biomaterials based on nanocellulose are commonly prepared by combining nanocellulose with other antimicrobial agents (frequently using silver) using either chemical or physical approaches. The antibacterial assays have confirmed the efficient antibacterial activity of several nanocellulose-based antimicrobial biomaterials. Modified cellulose-based nanofibrils have been effective to inhibit bacterial growth (in both liquid medium and agar plates), and can kill several logs of microbial cells. Moreover, evaluation of wound healing *in vivo* and *in vitro* has shown that nanocellulose-based antimicrobial hydrogel dressings possess good biocompatibility, remarkable antibacterial efficacy, and can hasten the time course of wound healing. Cellulose-based nanofibrils can act as a bio-composite for antibacterial applications, as well as drug carriers, packaging materials, and wound dressing materials. This approach using cellulose-based nanocomposite hydrogels with multi-component construction could provide innovative wound dressings with long-lasting antimicrobial activity.
